# A Rare Case of Subclavicular Cystic Lymphangioma in Adults Adhering to Axillary Vessels

**DOI:** 10.7759/cureus.47341

**Published:** 2023-10-19

**Authors:** Hafedh Daly

**Affiliations:** 1 Cardiovascular Surgery, Faculty of Medicine of Monastir, Monastir, TUN

**Keywords:** surgery, axillary vessels, adult, subclavicular, cystic lymphangioma

## Abstract

Cystic lymphangiomas are rare and benign lymphatic malformations. They often sit in the cervico-facial or axillary region. We report the case of a 31-year-old patient admitted for a left subclavicular mass symptomatic of pain on arm abduction. The CT scan of the chest suggests a cystic lymphangioma adhering to the axillary vascular pedicle. The patient had a complete resection of this mass with a simple postoperative course.

## Introduction

Cystic lymphangiomas are rare, hemodynamically inactive, benign, and mature lymphatic malformations consisting of abnormal lymphatic vessels and cysts of various sizes and shapes [1-3]. They occur in children, with more than 90% of cases seen in patients under two years of age while only 7% occur in adults [4,5]. The most commonly used classification divides them into microcystic, macrocystic, and mixed lesions [1,6]. Macrocystic lesions are cystic spaces with a volume of more than 2 cm³, microcystic lesions are composed of elements less than 2 cm³, and mixed lesions contain both types of cysts [1,7].

Some authors prefer to classify them as capillary lymphangiomas, cavernous lymphangiomas, and cystic hygromas [1,3,8]. They are located at the neck in 75% of cases and in the axillary region in 20% of cases. Mediastinal, retroperitoneal, and pelvic localization are rare [2,3].

We report a rare case of left subclavicular cystic lymphangioma in a North African man.

## Case presentation

A 31-year-old man with no significant pathological history was admitted for surgical evaluation of a left subclavicular mass that caused pain upon upper limb abduction.

**Figure 1:**
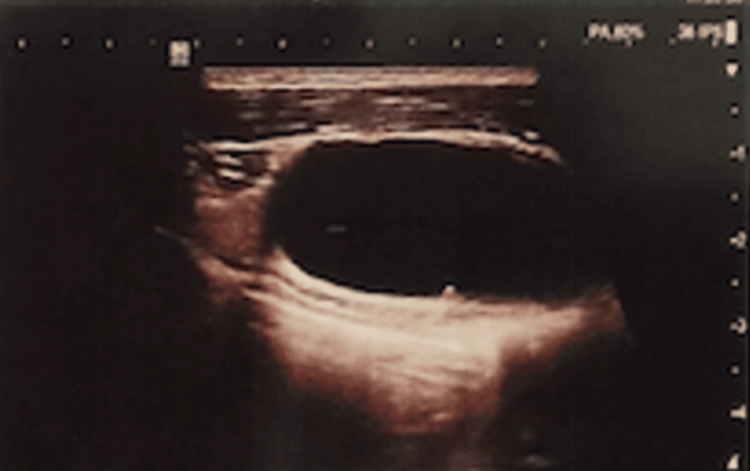
Ultrasound of a cyst with regular walls and transonic content

**Figure 2:**
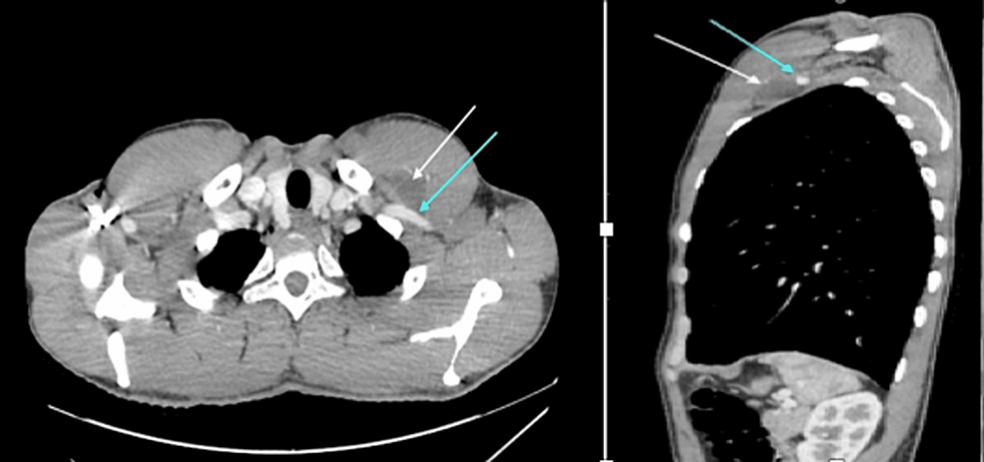
Chest CT scan showed deep cystic formation of the anterior and upper left chest wall. The lesion is anterior and inferior to the left axillary artery and in contact with the left first rib.

**Figure 3:**
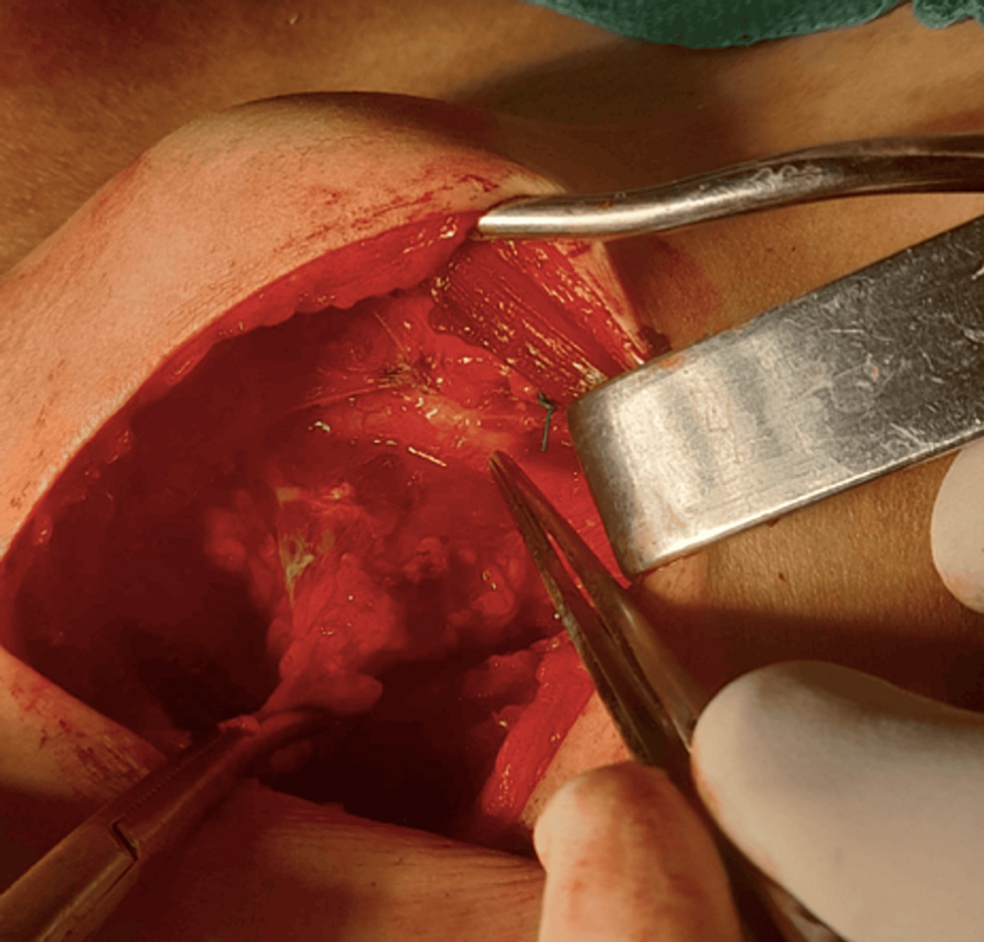
Intraoperative view showing cystic lymphangioma adhering to axillary vessels

**Figure 4:**
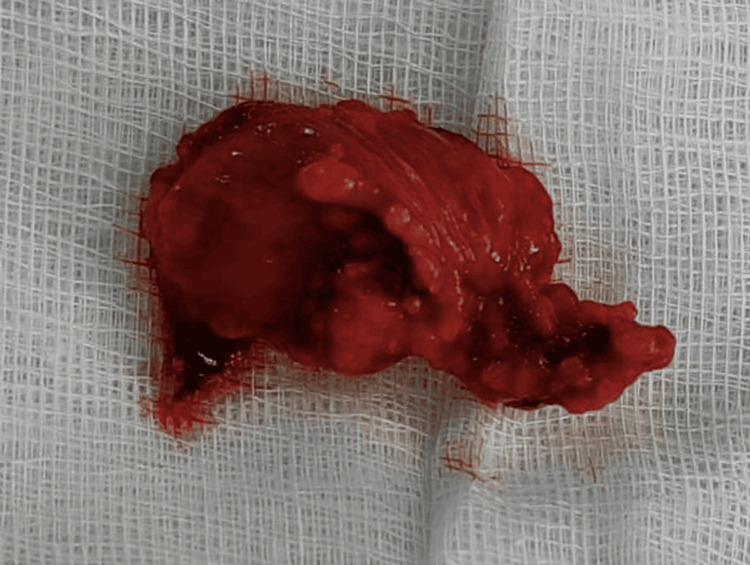
The specimen with a cystic appearance

**Figure 5:**
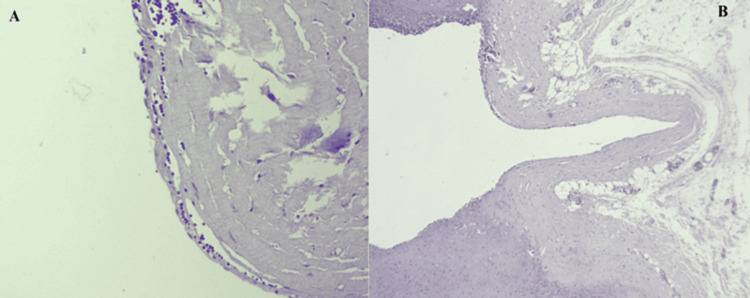
Histological appearance of the cystic lymphangioma showing a fibrous wall (A) and a simple and non-atypical endothelial coating associated with a subepithelial lymphocytic infiltrate (B)

On physical examination, slight asymmetry was detected in the left subclavicular region, with the presence of a firm mass. Ultrasound revealed a 41 by 16 mm left subclavicular upper chest wall cyst with regular walls and transonic content located below the pectoralis major muscle and medial to the upper part of the pectoralis minor muscle (Figure 1). This cystic mass was in contact above the normal vascular anatomy. The chest CT scan showed a deep cystic formation of the anterior and upper left chest wall in a retro-pectoral and pre-costal area with a 43 mm long axis. The noted abnormality was anterior and inferior to the left axillary artery and in contact with the anterior arch of the left first rib (Figure 2) without lysis or condensation. This aspect was initially suggestive of a cystic lymphangioma. The patient was operated on via a left subclavicular approach. Meticulous dissection allowed complete excision of this cystic mass, which was intimately adherent to the axillary artery and vein (Figures 3, 4). The postoperative course was uneventful with no sign of recurrence at six months postoperatively. Histological examination confirmed the diagnosis of a subclavicular cystic lymphangioma. On microscopy, numerous inter-communicating cavities of a lymphatic nature were identified in fibro-adipose connective tissue. These cavities were lined by a flattened endothelium containing lymph and lymphocytes. In addition, the connective tissue was slightly modified. In the wall, ectatic lymph cavities and a small benign lymph node were found (Figure 5).

## Discussion

Cystic lymphangiomas are rare benign lymphatic malformations resulting from lymphatic dilation with endothelial linings. It is a benign, slow-growing tumor [9,10] caused by congenital weakness in the wall, with blockage of lymphatic channels or proliferation of lymphatic vessels [9,11].

Adult presentation of lymphangioma is extremely rare [12]. Our case is novel because this cystic lymphangioma had an intimate anatomical relationship with the left axillary artery and vein.

Hematoma, abscess, lymphocele, and soft tissue sarcomas constitute the differential diagnosis of cystic lymphangioma [13].

Ultrasound is the first examination to be performed in order to clarify the cystic or solid nature of the mass. Cystic lymphangioma appears as a multilocular mass, separated by septa, often anechoic, sometimes hypoechoic, or hyperechoic if there is infection, calcifications, or hemorrhage [13,14].

However, the use of a CT scan provides a means to differentiate a cystic lymphangioma from other soft tissue masses such as sarcomas, as well as guide surgery by studying its extent and anatomical relationships. Cystic lymphangioma appears on CT as a multilocular cystic mass with smooth septa, which is enhanced with contrast product [13,15]. Therefore, a CT scan offers important findings to arrive at a diagnosis.

Magnetic resonance imaging (MRI) is currently an essential imaging study to arrive at a diagnosis [16,17]. It makes it possible to assess the extent of involvement with surrounding tissues to better guide surgery. On MRI, lymphangiomas are hypointense or isointense relative to muscle on T1-weighted and hyperintense on T2-weighted [17,18]. In our case, this examination was not available in our health facility.

Several therapeutic modalities have been described in the literature. Sclerotherapy, laser, and cyst aspiration are available [19,20], but surgical excision of the mass while preserving adherent or adjacent vessels or nerve pathways remains the standard therapeutic option [13,19,20].

## Conclusions

The development of subclavicular cystic lymphangioma in adults is extremely rare. The present case is novel because of its anatomical relationships with the axillary artery and vein. Although unusual in adults, cystic lymphangioma should be considered in the presence of any subclavicular swelling. The different imaging modalities are necessary to eliminate other differential diagnoses. The diagnostic confirmation relies on histopathological assessment. Surgical excision constitutes the therapeutic option of choice.
